# Inoculation of tomato with a plant growth-promoting rhizobacteria enhances basal and wound-induced ROS levels

**DOI:** 10.1093/plphys/kiaf054

**Published:** 2025-02-18

**Authors:** Lidia S Pascual, María Ángeles Peláez-Vico, Aurelio Gómez-Cadenas, Sara I Zandalinas, Ron Mittler

**Affiliations:** Department of Biology, Biochemistry and Environmental Sciences, University Jaume I, Av. de Vicent Sos Baynat, s/n, Castelló de la Plana 12071, Spain; Division of Plant Science and Technology, College of Agriculture Food and Natural Resources, Christopher S. Bond Life Sciences Center, University of Missouri, 1201 Rollins St., Columbia, MO 65211, USA; Department of Biology, Biochemistry and Environmental Sciences, University Jaume I, Av. de Vicent Sos Baynat, s/n, Castelló de la Plana 12071, Spain; Department of Biology, Biochemistry and Environmental Sciences, University Jaume I, Av. de Vicent Sos Baynat, s/n, Castelló de la Plana 12071, Spain; Division of Plant Science and Technology, College of Agriculture Food and Natural Resources, Christopher S. Bond Life Sciences Center, University of Missouri, 1201 Rollins St., Columbia, MO 65211, USA

Dear Editor,

Rapid systemic signaling, mediated by the reactive oxygen species (ROS), calcium, and electric waves, is a highly dynamic process essential for plant acclimation to stress (Kollist et al. 2019; Zandalinas et al. 2020a; Mittler et al. 2022; Suda and Toyota 2022). It has been shown to transmit a systemic signal within minutes from a stressed or stimulated plant tissue to the rest of the plant via a cell-to-cell communication pathway that requires the coordinated function of multiple proteins and tissues (Zandalinas et al. 2020b; Fichman et al. 2022). Although rapid systemic signaling has been extensively studied in recent years, how this process is affected by the interactions of the plant with its microbiome is poorly defined. Plant growth-promoting rhizobacteria (PGPR) constitute a diverse group of bacteria that primarily interact with the plant root system and promote plant growth, as well as in some cases resilience to different abiotic stresses (Batool et al. 2024; Berruto and Demirer 2024; He et al. 2024). To study the impact of plant–microbe interactions on systemic signaling we chose the tomato (*Solanum lycopersicum*)–*Pseudomonas putida* KT2440 interaction system (Costa-Gutierrez et al. 2022). *P. putida* was previously reported to enhance tomato and citrus (*Citrus macrophylla*) growth and resilience to salinity stress (Shen et al. 2012; Vives-Peris et al. 2018; confirmed here in Fig. 1A and Supplementary Fig. S1). To simplify the complex root microbiome–plant interactions, we chose to use a simple binary plant–microbe interaction of sterile soil with or without *P. putida* (Supplementary Methods and Table S1). This design allowed us to examine the effects of *P. putida* inoculation on rapid systemic signaling in tomato in the relative absence of other competing or interacting root microorganisms (Russ et al. 2023; Jones et al. 2024). As shown in Fig. 1B, compared to noninoculated plants, plants inoculated with *P. putida* maintained an overall higher basal levels of ROS in their local and systemic leaves (detected with the broad-spectrum ROS dye 2′,7′-dichlorofluorescin diacetate; Fichman and Mittler 2021). Despite the high basal levels of ROS in *P. putida* inoculated plants, both local and systemic levels of ROS were further elevated in response to wounding (Fig. 1B). While the overall increase (in fold change) was relatively similar between inoculated and noninoculated plants, the overall ROS levels of inoculated plants were higher both before and after wounding. Similar findings were obtained when whole plant levels of H_2_O_2_ were measured using the H_2_O_2_-specific dye Peroxy Orange 1 (Fichman et al. 2022), or when H_2_O_2_ levels were measured in leaf extracts using the Amplex Red method (Fig. 1C; Fichman et al. 2022). In agreement with these findings, compared to uninoculated plants, the steady-state level of transcripts encoding the ROS-generating enzyme respiratory burst oxidase homolog 1 (*SlRBOH1*; Zheng et al. 2024) was significantly higher in wounded or unwounded *P. putida* inoculated plants (Fig. 1D).

**Figure 1.**
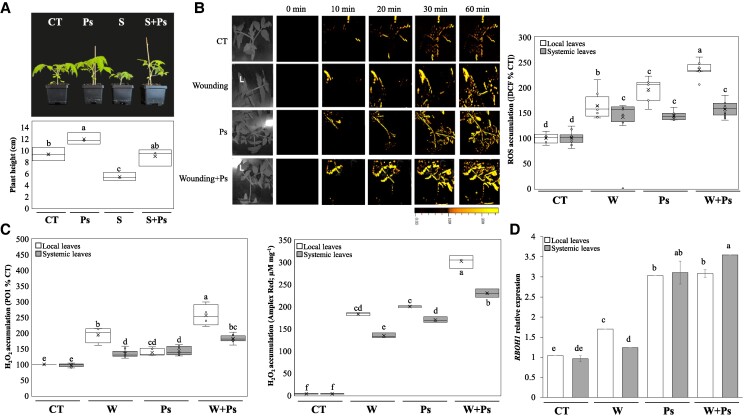
The impact of PGPR on basal and wound-induced ROS levels in tomato. **A)** Representative images (top) and a bar graph (bottom) showing that inoculation with *P. putida* KT2440 enhances plant growth and resilience to salinity in tomato. **B)** Representative images (left) and a bar graph (right) showing the effect of *P. putida* inoculation on overall ROS accumulation in local and systemic leaves of tomato plants maintained under controlled conditions or subjected to wounding (at the local leaf). Graph represents measurements at 60 min post-wounding. **C)** Bar graphs showing the effect of *P. putida* inoculation on H_2_O_2_ accumulation in local and systemic leaves of wounded and unwounded tomato plants 60 or 30 min post-wounding. Left, results from whole plant imaging with Peroxy Orange 1 (60 min); right, results for Amplex Red measurements in leaf extracts (30 min). **D)** Steady-state transcript levels of respiratory burst homolog 1 (*SlRBOH1*) in local and systemic tomato leaves from inoculated and noninoculated plants kept under controlled conditions or wounded at their local leaf. Graph represents measurements at 10 min post-wounding. Box plot graphs are presented with mean as *X* ± SE; median is the line in the box and box borders are 25th and 75th percentiles; whiskers are the 1.5 interquartile range. Statistical analysis was performed with the Statgraphics Plus v5.1. software by 2-way analysis of variance followed by Tukey’s post hoc test (different letters denote statistical significance at *P* < 0.05; *n* = 10). CT, control; DCF, dichlorodihydrofluorescein; PGPR, plant growth-promoting rhizobacteria; Ps, *Pseudomonas putida* KT2440 (*P. putida*); *RBOH1*, respiratory burst homolog 1; ROS, reactive oxygen species; S, salinity; W, wounding.

To further determine the impact of *P. putida* inoculation on rapid systemic signaling, we measured the calcium wave in inoculated and noninoculated plants following wounding (using the same setup shown in Fig. 1). For these measurements, we used the cytosolic calcium dye Fluo-4-AM (Fichman and Mittler 2021; Fichman et al. 2022). As shown in Fig. 2A, like ROS and H_2_O_2_ (Fig. 1), plants inoculated with *P. putida* displayed constitutively elevated levels of cytosolic calcium in the absence of wounding. In response to wounding, both inoculated and noninoculated plants displayed an enhanced calcium response that, like the results with ROS (Fig. 1), was not different in its extent from each other (Fig. 2A). As calcium and ROS levels are linked in cells (Waszczak et al. 2018; Smirnoff and Arnaud 2019; Mittler et al. 2022; Suda and Toyota 2022), the higher calcium levels, in the presence of higher levels of RBOH1 (Zheng et al. 2024), could cause higher levels of ROS in *P. putida*–inoculated plants even in the absence of wounding (Figs. 1 and 2A). The findings that both ROS and calcium levels are elevated in inoculated plants (Figs. 1 and 2A) prompted us to test the steady-state level of wound response transcripts in inoculated and noninoculated plants in the presence or absence of wounding. As shown in Fig. 2B, compared to noninoculated and nonwounded plants, inoculated and nonwounded plants displayed an elevated steady-state level of several different wound and ROS response transcripts (i.e. *SlPI1*, protease inhibitor 1, enhances resistance to stress by promoting ROS accumulation, Thole et al. 2009; *SlJAZ2*, jasmonate ZIM-domain protein 2, involved in jasmonic acid and ROS signaling, Wang et al. 2020; *SlFAD7*, fatty acid desaturase 7, involved in lipid and ROS signaling, Nishiuchi et al. 1997; *SlWIND1*, wound inducible dehydration-responsive element-binding protein 1, cell dedifferentiation regulator, Yang et al. 2024; *SlPORK1*, protein of related kinase 1, modulates ROS and programmed cell death in response to wounding, Yang et al. 2024; and *SlAPX1*, ascorbate peroxidase 1, H_2_O_2_-scavenging enzyme, Smirnoff and Arnaud 2019). While the steady-state level of some of these transcripts was unchanged following wounding in *P. putida* inoculated plants (e.g. *SlPI1* in systemic leaves), the steady-state level of other transcripts declined (e.g. *SlFAD7* in local leaves; Fig. 2B). The findings presented in Fig. 2B suggest that, in addition to high ROS and calcium levels, *P. putida*–inoculated plants could be primed for a wound response, even in the absence of wounding. Further studies are needed to determine the biological significance of the transcript expression changes shown in Fig. 2B, their relevance to regular wound responses (that sometimes involve transient expression of some of these transcripts), and their association with ROS levels in PGPR-inoculated plants. Further studies are also needed to determine the molecular mechanisms that control the elevated calcium and ROS levels in *P. putida*–inoculated tomato plants, as well as to define whether *P. putida* induces in plants a state of induced systemic resistance.

**Figure 2.**
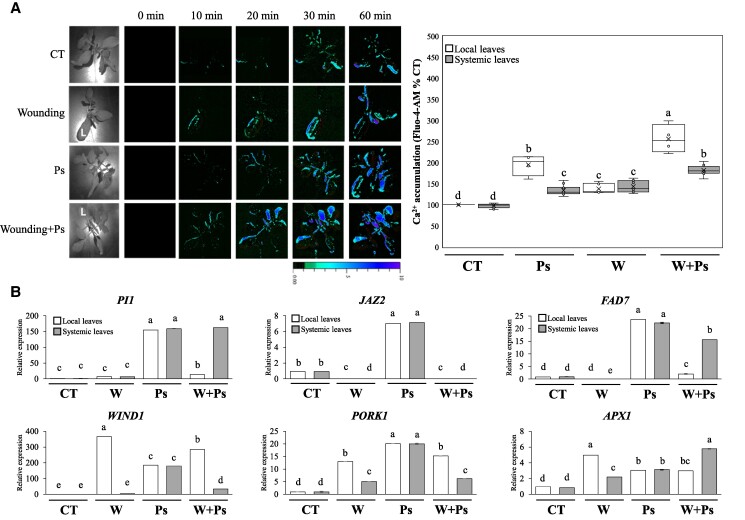
The impact of PGPR on calcium and wound response transcript expression. **A)** Representative images (left) and a bar graph (right) showing the effect of *P. putida* KT2440 (*P. putida*) inoculation on cytosolic calcium accumulation measured with Fluo-4-AM imaging in local and systemic leaves of tomato plants maintained under controlled conditions or subjected to wounding (at the local leaf). Graph represents measurements at 60 min post-wounding. **B)** Steady-state transcript levels of *SlPI1*, *SlJAZ2*, *SlFAD7*, *SlWIND1*, *SlPORK1*, and *SlAPX1* in local and systemic tomato leaves from *P. putida* inoculated and noninoculated plants kept under controlled conditions or wounded on their local leaf. Graph represents measurements at 10 min post-wounding. Box plot graphs are presented with mean as *X* ± SE; median is the line in the box and box borders are 25th and 75th percentiles; whiskers are the 1.5 interquartile range. Statistical analysis was performed with the Statgraphics Plus v5.1. software by 2-way analysis of variance followed by Tukey’s post hoc test (different letters denote statistical significance at *P* < 0.05; *n* = 10). *APX1*, ascorbate peroxidase 1; CT, control; *SlFAD7*, fatty acid desaturase 7; *SlJAZ2*, jasmonate ZIM-domain containing 2; PGPR, plant growth-promoting rhizobacteria; *PI1*, protease inhibitor 1; *PORK1*, protein of related kinase 1; Ps, *Pseudomonas putida* KT2440 (*P. putida*); *WIND1*, wound inducible dehydration-responsive element-binding protein 1; W, wounding.

Taken together, our findings reveal that inoculation of tomato with *P. putida* causes enhanced accumulation of calcium, ROS, and different wound response transcripts without negatively impacting plant growth or rapid systemic signaling responses to wounding (Figs. 1 and 2; enhanced ROS accumulation in PGPR-inoculated plants was also shown by Audenaert et al. 2002 and Peleg-Grossman et al. 2012, and no, or positive, effects of *P. putida* on plant growth during stress were also shown by Batool et al. 2024, Berruto and Demirer 2024, and He et al. 2024). This is an interesting finding, as it is generally thought that higher levels of ROS, and/or constitutive induction of different defense and acclimation responses, will suppress growth (e.g. Sies and Jones 2020; Zhang et al. 2024). The plant/soil microbiome could therefore influence the overall levels of oxidants in plants, but this does not stop further responses when stress is applied. Accordingly, we show that systemic signaling can still function even in PGPR-inoculated plants that are already primed to have high ROS, calcium, and transcript expression levels (Figs. 1 and 2). Further studies are of course needed to test the impact of a higher level of complexity in root and leaf microbiomes (e.g. that induced by different communities of microbes) on rapid systemic responses of plants to stress and the overall process of plant stress acclimation. We hope that this letter will promote such studies as they are essential to our understanding of how the plant microbiome impacts plant health in nature and the field (Finkel et al. 2017; Russ et al. 2023; Jones et al. 2024).

## Supplementary Material

kiaf054_Supplementary_Data

## Data Availability

The data that support the findings of this study are available in the text, figures, and supplemental material of this article.

## Supplementary data

The following materials are available in the online version of this article.

**Supplementary Table S1.** Composition of M8 medium.

**Supplementary Figure S1.** Representative image of tomato plants 10 days post-inoculation with *P. putida*, prior to being subjected to wounding and analysis.

## Dive Curated Terms

The following phenotypic, genotypic, and functional terms are of significance to the work described in this paper:

APX1 Gramene: AT1G07890
APX1 Araport: AT1G07890
