# Supplementary material for: Inoculation of tomato with a plant growth-promoting rhizobacteria enhances basal and wound-induced ROS levels
Source: Plant Physiol. 2025 Feb 18;197(2):kiaf054. doi: 10.1093/plphys/kiaf054 (PMC11834975; doi:10.1093/plphys/kiaf054)
Supplement: kiaf054_Supplementary_Data [file kiaf054_supplementary_data.pdf]

Supplementary Material for:

Inoculation of tomato with a plant growth-promoting rhizobacteria enhances basal and wound-induced ROS levels

Lidia S Pascual[1], María Ángeles Peláez-Vico[2], Aurelio Gómez-Cadenas[1], Sara I. Zandalinas[1], and Ron Mittler[2]

[1]Department of Biology, Biochemistry and Environmental Sciences, University Jaume I. Av. de Vicent Sos Baynat, s/n, Castelló de la Plana, 12071, Spain.

[2]Division of Plant Science and Technology, College of Agriculture Food and Natural Resources, Christopher S. Bond Life Sciences Center, 1201 Rollins St., University of Missouri, Columbia, MO 65211, USA.

*Corresponding author (mittlerr@missouri.edu)

Contains:

Supplementary Material and Methods

Figure S1

Table S1

Supplementary Material and Methods

Plant growth and salinity stress conditions

Moneymaker tomato seeds (Clemente Viven, Semillas Clemente S.A., Vitoria, Álava, Spain) were used for *Pseudomonas putida* inoculation and experiments. Seeds were sown in trays filled with a growing medium consisting of peat moss, perlite, and vermiculite in a ratio of 80:10:10. Following germination, seedlings were washed with water and transplanted into 8-cm diameter pots, filled with a sterile mixture of autoclaved peat moss and perlite (2:1). Plants were maintained under controlled conditions with a light intensity of 200 µmol photons m^{-2} s^{-1}, following an8-hour light/16-hour dark photoperiod, and average temperature of 25.0°C. To ensure proper nutrition, plants were watered twice a week with half-strength Hoagland solution and once a week with water, as previously described in (Pascual et al., 2023). To induce salinity stress, four-week-old

tomato plants were treated with 120 mM NaCl twice a week using half-strength Hoagland solution and once a week with water containing the salinity stressor for two weeks. Images and measurements were taken 15 days post treatment initiation.

Bacterial growth and plant inoculation

Pseudomonas putida KT2440 (Vives-Peris et al., 2018) was cultured in flasks containing lysogeny broth (LB) liquid medium (Bertani, 1951), supplemented with 50 mg/mL chloramphenicol (Franklin and Aldridge, 1976). Cultures were maintained at 25ºC in an orbital shaker at 120 rpm. Bacteria were centrifuged at 10,000 g for 15 minutes, and the resulting pellet was resuspended in M8 minimal medium (Köhler et al., 2000; Table S1). The bacterial culture was then adjusted to an initial optical density (OD) of 0.05 at 660 nm using a Genesys 10UV spectrophotometer (Thermo Scientific, Massachusetts, USA). Ten days prior to the wound stress treatments, inoculated tomato plants were soil irrigated with *P. putida* KT2440; and control plants, not inoculated with bacteria, were soil irrigated with the same volume of M8 minimal medium.

Wounding and IVIS imaging of ROS and calcium levels

Tomato plants were fumigated with 50 µM H2DCFDA (Ex./Em. 480 nm/520 nm; Millipore-Sigma) for general ROS imaging, 4.5-mM Fluo-4-AM (Ex./Em. 494 nm/506 nm; Becton, Dickinson and Company, Franklin Lakes, NJ, USA) for calcium imaging, or 100-µM Peroxy orange 1 (PO1, Ex./Em. 540 nm/565 nm; Millipore-Sigma, St. Louis, MO, USA) for H_2O_2 imaging, in 0.05 M phosphate buffer pH 7.4 with 0.01% Silwet L-77. Fumigation was carried out for 30 min using nebulizers (Punasi Direct, Hong Kong, China) in a glass container. Following fumigation, local wounding was applied to a single tomato leaflet of an upper leaf, as described by Fichman and Mittler (2021). Tomato plants were imaged 60 minutes post-wounding for ROS/Ca^{2+}/H_2O_2 accumulation using the IVIS Lumina S5 platform as previously described in Fichman et al. (2022).

Measurements of H_2O_2 levels in leaf extracts

Wounded and unwounded leaves were obtained from inoculated and non-inoculated tomato plants 30 minutes post wounding, flash frozen in liquid nitrogen, ground to fine powder, and subjected to an Amplex Red assay, as described by Fichman et al, (2022). A calibration curve of H_2O_2 and tissue powder weight were used to calculate H_2O_2 levels as described by Fichman et al, (2022).

RNA isolation and RT-qPCR

Wounded and unwounded leaves were sampled from inoculated and non-inoculated tomato plants, 10 minutes post wounding, and flash frozen in liquid nitrogen, as described by Fichman et al, (2022). RNA was extracted from frozen leaf tissue using an RNeasy Mini kit (Qiagen, Hilden, Germany) following the manufacturer's instructions. Total RNA concentration and purity were determined using a NanoDrop One spectrophotometer (Thermo Scientific, Massachusetts, USA). Reverse transcription was performed from 1 µg of total RNA using PrimerScript RT reagent with

oligo (dT) primer (Takara Bio Inc., Kusatsu, Japan). Relative expression analysis by RT-qPCR was performed in a CFX Connect Real-Time PCR system (Bio-Rad, CA, USA). The reaction mixture contained 1 µL of cDNA, 5 µL of SYBR Green (Applied Biosystems, CA, USA) and 1 µM of each gene-specific primer pair in a final volume of 10 µL. The thermal profile used to analyze the relative gene expression consisted of 10 min at 95°C for pre-incubation, followed by 40 cycles of: 10 s at 95°C for denaturation, 10 s at 60°C for annealing, and 20 s at 72°C for extension. Amplicon specificity of the PCR reaction was evaluated by the presence of a single peak in the dissociation curve after the amplification steps. The expression levels of all genes were normalized against the expression of two endogenous control genes (*Actin* [*Solyc03g078400*] and *EF1α* [*Solyc06g005060*]) based on previous housekeeping selection for tomato (Mascia et al., 2010) and the relative expression was calculated by using REST (Pfaffl et al., 2002). For all genes studied, the reference for local samples was the expression value obtained for the control local conditions, whereas the reference for systemic samples was the expression value obtained for the control systemic conditions. Reference conditions were set as 1. Three technical replicates were analyzed on each biological replicate.

Statistical Analysis

All experiments were repeated at least three times with three biological repeats. Box plot graphs are presented with mean as X ± SE; median is the line in the box and box borders are 25th and 75th percentiles; whiskers are the 1.5 interquartile range. Statistical analysis was performed with the Statgraphics Plus v5.1. software (Statistical Graphics Corp., Herndon, VA, United States) by two-way analysis of variance (ANOVA) followed by Tukey post hoc test (different letters denote statistical significance at $P < 0.05$).

References for Supplementary Material and Methods

Bertani G (1951) Studies on lysogenesis. I. The mode of phage liberation by lysogenic *Escherichia coli*. J Bacteriol **62**: 293–300

Fichman Y, Mittler R (2021) Integration of electric, calcium, reactive oxygen species and hydraulic signals during rapid systemic signaling in plants. Plant J **107**: 7–20

Fichman Y, Zandalinas SI, Peck S, Luan S, Mittler R (2022) HPCA1 is required for systemic reactive oxygen species and calcium cell-to-cell signaling and plant acclimation to stress. Plant Cell **34**: 4453–4471

Franklin RA, Aldridge A (1976) Studies on the metabolism of meptazinol, a new analgesic drug. Br J Clin Pharmacol **3**: 497–502

Köhler T, Curty LK, Barja F, van Delden C, Pechère JC (2000) Swarming of *Pseudomonas aeruginosa* is dependent on cell-to-cell signaling and requires flagella and pili. J Bacteriol **182**: 5990–5996

Mascia T, Santovito E, Gallitelli D, Cillo F (2010) Evaluation of reference genes for quantitative

reverse-transcription polymerase chain reaction normalization in infected tomato plants. Mol Plant Pathol **11**: 805–816

Pascual LS, Mittler R, Sinha R, Peláez-Vico MÁ, López-Climent MF, Vives-Peris V, Gómez-Cadenas A, Zandalinas SI (2023) Jasmonic acid is required for tomato acclimation to multifactorial stress combination. Environ Exp Bot **213**: 105425

Pfaffl MW, Horgan GW, Dempfle L (2002) Relative expression software tool (REST©) for group-wise comparison and statistical analysis of relative expression results in real-time PCR. Nucleic Acids Res **30**: e36**Vives-Peris V, Gómez-Cadenas A, Pérez-Clemente RM** (2018) Salt stress alleviation in citrus plants by plant growth-promoting rhizobacteria Pseudomonas putida and Novosphingobium sp. Plant Cell Rep **37**: 1557–1569

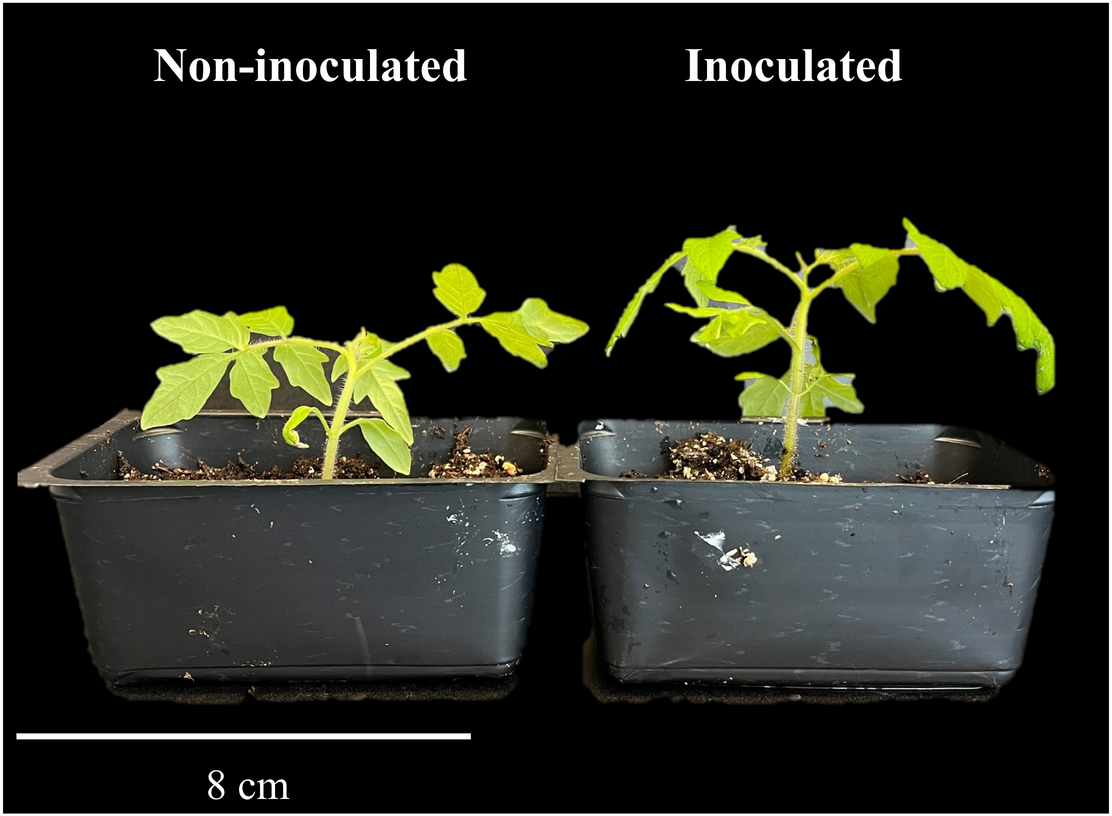

Supplementary Figure S1. Representative image of tomato plants 10 days post inoculation with *P. putida*, prior to being subjected to wounding and analysis.

Table S1. Composition of M8 medium

Compounds	V (1L)
Disodium hydrogen phosphate (Na_2HPO_4)	7 g
Potassium dihydrogen phosphate (KH_2PO_4)	3 g
Sodium chloride (NaCl)	0.5 g
Water	Up to 1L
